# Isolation, Characterization, and Anti-Inflammatory Effects of *Carthamus tinctorius* L. Leaf-Derived Exosome-like Nanoparticles in ETEC-Challenged IPEC-J2 Cells

**DOI:** 10.3390/foods15142417

**Published:** 2026-07-08

**Authors:** Yongmei Luo, Kang Ma, Xiaoyan Wang, Kangjun Fan, Hongzao He, Zhaojun Wei, Xueli Hu, Jiao Liu, Rui Qin, Hong Liu

**Affiliations:** 1 Hubei Provincial Key Laboratory for Protection and Application of Special Plant Germplasm in Wuling Area of China, College of Life Sciences, South-Central Minzu University, Wuhan 430074, China; yongmeiluo5316@163.com (Y.L.); mvp2003@126.com (K.M.);; 2 Guizhou Academy of Testing and Analysis, Guiyang 550081, China; 3 School of Biological Science and Engineering, North Minzu University, Yinchuan 750021, China; 4 Industrial Crop Research Institute, Yunnan Academy of Agricultural Sciences, Kunming 650205, China

**Keywords:** plant-derived exosome-like nanoparticles (PELNs), *Carthamus tinctorius* L., anti-inflammatory activity, IPEC-J2 cells

## Abstract

Safflower (*Carthamus tinctorius* L.) is a cash crop grown worldwide. Its seeds and flowers are primarily processed for edible oil and medicinal raw materials, while stems and leaves are discarded as agricultural waste. Recently, plant-derived exosome-like nanoparticles (PELNs) have gained growing research interest in food nutrition owing to their exceptional biocompatibility and relatively low cost for large-scale production. This study aimed to explore the potential functional value of safflower leaf agricultural waste by isolating safflower-derived PELNs. Firstly, a protocol was established for the isolation and purification of safflower (*Carthamus tinctorius* L.)-derived exosome-like nanoparticles (Ct-ELNs) from safflower leaves using sucrose density gradient ultracentrifugation. NTA analysis revealed that the 30–45% sucrose fraction enriched with Ct-ELNs exhibited the most uniform particle size, highest particle concentration, and optimal purity. Transmission electron microscopy confirmed typical PELNs ultrastructure: disc- or cup-shaped vesicles surrounded by bilayer lipid membranes. FM4-64 fluorescence suggests time-dependent association and likely cellular uptake of labeled Ct-ELNs by IPEC-J2 cells. Cellular assays demonstrated that Ct-ELNs elevated IPEC-J2 cell metabolic activity under matched protein-normalized dosing conditions. The 30–45% sucrose fraction showed the most favorable physicochemical profile and preliminary in vitro protective effects, including improved IPEC-J2 cell metabolic activity and modulation of Enterotoxigenic *Escherichia coli* (ETEC)-induced inflammation-related gene expression. Overall, this study established an optimized isolation protocol for Ct-ELNs derived from safflower leaves. These data indicate that safflower-derived Ct-ELNs confer preliminary cytoprotective transcriptional regulatory effects on intestinal epithelial cells under in vitro culture conditions.

## 1. Introduction

Safflower (*Carthamus tinctorius* L.) is an annual oil crop belonging to the Asteraceae family. It is extensively cultivated owing to its substantial economic value, which encompasses medicinal applications (utilizing its florets), oil extraction (from its seeds), and dye production (from its florets) [1,2,3]. In the realm of traditional medicine, safflower florets have long been employed for the treatment of angina pectoris, stroke, gynecological disorders, and hypertension. Studies have demonstrated that safflower inflorescences are abundant in bioactive constituents dominated by flavonoids, which exert multiple pharmacological effects including antithrombotic, anti-inflammatory, anticarcinogenic, and hypoglycemic activities [4,5]. Historically, the orange-red pigments extracted from safflower petals have been widely applied in food coloring and textile dyeing. In recent years, safflower cultivation has expanded significantly, largely due to increasing demand for its seeds which are valued for their content of ω 3 fatty acids and other nutrients. Safflower seed oil, as a premium edible oil, is commonly used in culinary practices, salad dressings, and various processed foods [4].

Besides the most frequently used parts, i.e., seeds and florets, studies have also reported that the leaves of safflower have laxative, appetitive, and diuretic effects and are therefore useful for urethritis and ophthalmopathy [6]. Safflower leaves are non-toxic and have been eaten as a vegetable in many countries [7]. For instance, in India, Pakistan, and Burma, young leaves from safflowers are boiled and consumed as a vegetable side dish with curry or rice [8]. A more recent study showed that safflower leaves are cognitively beneficial food for preventing and alleviating Alzheimer’s disease-related dementia [9]. Safflower leaves may be used to prepare functional tea products due to their potential bioactive functionalities [10]. The current industrial value of safflower is mainly based on the utilization of its seeds (for oil production and biodiesel) and flowers (for food colorants and flavoring agents), whereas the leaves are mostly discarded as agricultural waste [11,12,13]. Statistics from the Food and Agriculture Organization (FAO) (https://www.fao.org/faostat/zh/#compare, accessed on 15 May 2025) indicate that the maximum annual global cultivation area of safflower has reached 1,515,881 hectares, producing a large amount of waste leaves. Though safflower leaves have historically been recognized for their edibility and functionality, the development and utilization of safflower leaf components still remain significantly insufficient. The agricultural and food manufacturing industries produce large quantities of by-products each year. Treating these by-products simply as waste not only leads to the waste of biological resources but also exerts pressure on the environment. To date, a variety of bioactive and edible components, such as phenolic compounds, pectin, lipids, and dietary fiber have been successfully extracted from recycled by-products of the fruit and vegetable processing industry [14,15]. These recycled components are often used as food additives, thereby increasing the added value of primary raw materials. Among these recycled components, those with bioactive and nutritional properties have attracted particular attention from the food industry and researchers, owing to their potential added value in the production of functional foods, nutraceuticals, and cosmetics [16]. Notably, emerging studies have demonstrated that plant-derived exosome-like nanoparticles (PELNs) extracted from agricultural by-products and residues exhibit novel nutraceutical and therapeutic effects, attributed to their natural biological penetrability and the bioactive components they carry [17].

The application of PELNs has significant advantages in industrial fields. Firstly, PELNs can be obtained from a variety of plant raw materials, enabling efficient large-scale production [18]. In addition, the cell-derived components and phospholipid membranes inside PELNs endow them with excellent biocompatibility and safety, with low toxicity and minimal side effects [19]. Multiple studies have supported that PELNs, such as those from blueberries, strawberries, citrus peels, and *Andrographis paniculata*-derived plant extracellular vesicles exert significant protective effects against chronic intestinal inflammation by modulating inflammatory responses, cytokine secretion, and the expression of oxidative stress-related genes, thereby alleviating oxidative damage and regulating intestinal microbial homeostasis [20,21,22].

Porcine intestinal epithelial cells (e.g., the IPEC-J2 cell line) induced by Enterotoxigenic *Escherichia coli* (ETEC) are widely used as a classic in vitro model for intestinal inflammation [23,24]. The intestinal epithelial barrier serves as the first line of defense against intestinal pathogens [25]. In in vitro experiments, porcine intestinal epithelial cells (e.g., the IPEC-J2 cell line) represent a well-established model [24]. Derived from the jejunum of neonatal piglets, IPEC-J2 cells retain typical morphological characteristics, a distinct tight junction protein profile, and immune functions similar to primary epithelial cells and are capable of forming intact monolayers for intestinal barrier function assays [26,27]. The ETEC is a primary etiological agent responsible for post-weaning diarrhea in piglets [28]. Given its ability to induce IPEC-J2 cell apoptosis and disrupt epithelial barrier integrity, this strain is commonly employed as an inducer of intestinal injury in vitro [29,30,31]. In this study, the ETEC-induced IPEC-J2 inflammatory model was utilized. Although this model cannot fully recapitulate the full complexity of the in vitro intestinal microenvironment, including immune cell crosstalk, mucus barrier, gut microbiota, and systemic responses, it is recognized as a high-value and well-established in vitro system for exploring the cytoprotective and immunomodulatory potential of Ct-ELNs against intestinal epithelial injury and provides a reliable platform for dissecting the cellular effects of Ct-ELNs.

Recent studies have further shown that safflower-derived nanovesicles (CDNVs) isolated from safflower florets exhibit therapeutic effects on atherosclerosis through robust anti-inflammatory activity [32]. However, most current studies on safflower-derived nanovesicles primarily focus on florets and stigmas, which are constrained by low yield, high disease susceptibility, and unstable product quality. In contrast, safflower leaves are rich in flavonoids and various bioactive compounds and possess inherent anti-inflammatory and gastrointestinal regulatory properties [32]. Nevertheless, safflower leaves are commonly discarded as agricultural by-products, causing substantial resource waste. Distinct from previously reported flower-derived safflower nanovesicles, this study innovatively utilized waste safflower leaves as raw materials to fabricate Ct-ELNs, realizing the high-value utilization of agricultural green resources. Furthermore, the purification of plant extracellular vesicles currently faces prevalent technical limitations, including severe impurity contamination and non-uniform preparation protocols. To address these bottlenecks, sucrose density gradient ultracentrifugation was adopted in this study to achieve high-purity isolation of Ct-ELNs. Notably, the 30–45% sucrose fraction was screened as the optimal component, characterized by uniform particle size, high purity, and excellent biological activity. This finding provides a reliable and standardized enrichment strategy for the preparation of functional leaf-derived plant extracellular vesicles.

This study provides a new direction and experimental basis for the resource utilization of safflower leaves and lays a foundation for further exploration of the anti-inflammatory mechanism and industrial application of Ct-ELNs.

## 2. Materials and Methods

### 2.1. Isolation and Characterization of Ct-ELNs

**Figure 1:**
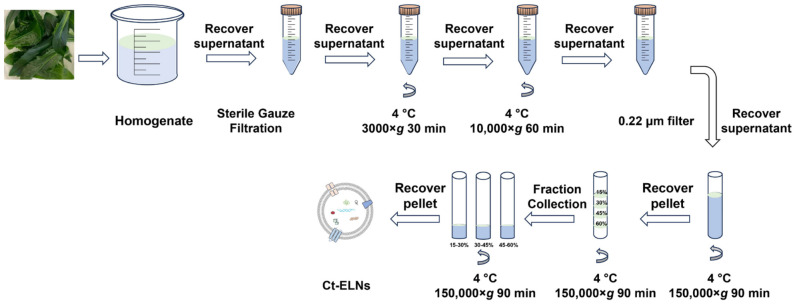
Schematic diagram of the isolation and purification process of Ct-ELNs from safflower leaves.

The present study utilized the Anhui-1 safflower germplasm, a purified cultivar independently developed by our laboratory in 2025 [2]. Corresponding voucher specimens have been retained in our laboratory. To guarantee experimental reproducibility, multiple independent batches of safflower materials were adopted for repeated trials. Batch-to-batch extraction reproducibility was systematically quantified, and key yield indicators were recorded, including the total particle number and total protein mass per gram of fresh leaves for each sucrose fraction. All quantitative data regarding extraction efficiency and inter-batch variation are summarized in Table S2. Plants were cultivated in a substrate containing vermiculite and nutrient soil at a ratio of 1:1 and grown in an incubator at 24 °C for 5 weeks until leaf harvest. Fresh leaves and phosphate-buffered saline (PBS, pH 7.2) were mixed at a ratio of 1 g:4 mL, followed by grinding for 30–60 s with a juice extractor as previously reported by Dad et al. [33]. Briefly, the mixture was blended at maximum speed for 3 min, paused for 1 min, and the cycle was repeated three times. The homogenate was filtered through double-layer sterile gauze. All samples used for Ct-ELNs extraction were fresh safflower leaves (Figure 1), and no fruit tissue was included in the preparation.

The resulting filtrate was subjected to sequential centrifugation at 3000× *g* for 30 min and 10,000× *g* for 60 min at 4 °C (Eppendorf 5810R, Hamburg, Germany). The harvested supernatant was filtered through a 0.22 μm membrane filter (Biosharp Life Sciences, Hefei, Anhui, China, model BS-PVDFL33-22-S) and then ultracentrifuged at 150,000× *g* for 90 min (Beckman Coulter Optima L-100XP, Brea, CA, USA). The obtained pellet was resuspended and further purified via sucrose density gradient ultracentrifugation. The gradient centrifugation was performed using an SW 41 Ti rotor and 13.2 mL thin-walled polypropylene (Beckman Coulter, Brea, CA, USA, model 331372) tubes. Each sucrose layer (15%, 30%, 45%, and 60%) was preloaded with a volume of 2 mL. After sample loading, the tube volume was supplemented with sterile PBS to full capacity. Following ultracentrifugation at 150,000× *g* for 120 min [34], each gradient fraction was individually aspirated using a sterile syringe and diluted with PBS to a final volume of 3 mL. To completely remove residual sucrose that may interfere with cell experiments, all collected fractions were re-ultracentrifuged at 150,000× *g* for 90 min. The final purified Ct-ELNs precipitate was resuspended in sterile PBS and stored at −80 °C until subsequent cell treatment experiments.

Nanoparticle tracking analysis (NTA). The particle size and concentration of Ct-ELNs were measured using NTA with ZetaView-PMX120-Z (Particle Metrix GmbH, Meerbusch, Germany, ZetaView® PMX-120-Z) and the corresponding software ZetaView (version 8.05.14 SP7, Particle Metrix GmbH, Meerbusch, Germany). Isolated exosome samples were appropriately diluted using 1 × PBS buffer (Beyotime Biotechnology, Shanghai, China) to measure the particle size and concentration. NTA measurement was recorded and analyzed at 11 positions. The ZetaView system was calibrated using 100 nm polystyrene particles. The laboratory ambient temperature was maintained at approximately 23 °C, and the sample cell temperature was set to 30 °C during measurement. Each sample was assayed in triplicate.

Transmission electron microscopy (TEM). The morphology analysis of Ct-ELNs was carried out by TEM (Jeol Ltd., Tokyo, Japan, JEM1400). A 20 μL aliquot of the resuspended sample was added dropwise onto a 200-mesh grid and incubated at room temperature for 10 min; then, the grids were negatively stained with 2% phosphotungstic acid for 3 min, and the remaining liquid was removed by filter paper. Subsequently, the grid was observed under a transmission electron microscope.

Protein quantification. The total protein concentration of Ct-ELNs was measured using the Enhanced BCA Protein Assay Kit (Beyotime Biotechnology, Shanghai, China) following the manufacturer’s protocol. After incubation at 37 °C in the dark for 30 min, the absorbance was measured at 562 nm using a Multi-functional Microplate Reader (Molecular Devices, San Jose, CA, USA, SpectraMax iD5). To denature the proteins in Ct-ELNs, radio immunoprecipitation assay (RIPA) lysis buffer supplemented with phenylmethylsulfonyl fluoride (PMSF; BestBio Biotechnology, Shanghai, China) was added, followed by heating at 95 °C for 10 min. The denatured proteins were then separated via sodium dodecyl sulfate-polyacrylamide gel electrophoresis (SDS-PAGE). Proteins in Ct-ELNs were visualized using the Fast Silver Stain Kit (Beyotime Biotechnology, Shanghai, China) in accordance with the kit’s instructions. All experiments were performed in triplicate.

### 2.2. Ct-ELNs Labeling

Fluorescent labeling of Ct-ELNs was carried out using the FM4-64 Fluorescent Cell Ligation Kit (Coolaber Science & Technology, Beijing, China) according to the manufacturer’s instructions. Briefly, Ct-ELNs were fluorescently labeled with the dye at a final concentration of 0.08% (*v*/*v*) and incubated at 37 °C in the dark for 15 min. Subsequently, 1 × PBS was added, and the sample was centrifuged for at 150,000× *g* 90 min at 4 °C to remove unbound free dye. Labeled Ct-ELNs pellets were resuspended in 200 μL PBS for further experiments.

### 2.3. IPEC-J2 Cell Culture Conditions

The IPEC-J2 cell line was obtained from Wuhan Key Cell Biotechnology Co., Ltd. (Wuhan, Hubei, China). Cells at passages 10 to 30 were used for all subsequent experiments. IPEC-J2 cells were cultured in DMEM/F12 medium (Servicebio Technology Co., Ltd., Wuhan, Hubei, China) supplemented with 10% (*v*/*v*) fetal bovine serum (Thermo Fisher Scientific, Shanghai, China) and 1% antibiotics (100 U/mL penicillin and 100 μg/mL streptomycin; Servicebio Technology Co., Ltd., Wuhan, Hubei, China). The cells were cultured in T75 culture flasks (Thermo Scientific™, Shanghai, China) and incubated in a carbon dioxide incubator at 37 °C with 5% CO_2_. Cell adhesion and growth were observed using an inverted biological microscope. The culture medium was replaced every 24 h. Cell passage was performed following the protocol described previously [35].

### 2.4. Cell Viability Assay

The CCK-8 assay was adopted to detect changes in cellular metabolic activity and relative viability. IPEC-J2 cells were seeded in 96-well plates at a final density of 1 × 10^4^ cells/well in 100 μL of culture medium and incubated overnight to promote cell adhesion to the plate surface. Potential interference from Ct-ELNs and co-extracted plant bioactive substances on the colorimetric reaction was ruled out by setting blank control wells containing only Ct-ELNs and CCK-8 reagent without seeded cells. When the cells reached 50% confluence, they were treated with Ct-ELNs at different concentrations (protein concentration: 5, 10, 20, 40, 80, 160, or 320 μg/mL) and co-incubated for 6, 12, 24, or 36 h [21,36]. At each time point, the cells were incubated with CCK-8 reagent, after which the absorbance was measured at 450 nm using a microplate reader. All experiments were performed in triplicate.

### 2.5. In Vitro Cellular Uptake Profiles of Ct-ELNs

To assess the uptake of Ct-ELNs by IPEC-J2 cells, labeled Ct-ELNs were co-incubated with fully adherent IPEC-J2 cells in DMEM/F12 medium at 37 °C with 5% CO_2_ for 6, 12, 24, and 36 h. After incubation, the cells were fixed with 4% paraformaldehyde (Servicebio Technology Co., Ltd., Wuhan, Hubei, China) for 10 min, followed by permeabilization with 0.1% Triton X-100 (Servicebio Technology Co., Ltd., Wuhan, Hubei, China) for 5 min. Subsequently, DAPI (Biosharp Life Sciences, Hefei, Anhui, China) and DIO (Beyotime Biotechnology, Shanghai, China) were added to label cell nuclei and cell membranes, respectively. Finally, the cells were mounted on coverslips, and images were captured using an Ultrahigh-Resolution Laser Scanning Confocal Microscope (Leica Microsystems GmbH, Wetzlar, Germany, Leica Stellaris 5). DAPI has a maximum excitation wavelength of 364 nm and a maximum emission wavelength of 454 nm. DIO maximum excitation wavelength: 484 nm. Maximum emission wavelength: 501 nm. FM4-64 maximum excitation wavelength: 543 nm Maximum emission wavelength: 640 nm.

### 2.6. Establishment of ETEC-Induced Epithelial Injury Model in IPEC-J2 Cells

We established an in vitro model of ETEC-induced epithelial injury in IPEC-J2 cells by optimizing bacterial culture duration (4, 6, and 8 h) and cell stimulation time (4, 6, and 8 h), with reference to the bacterial suspension concentration of 1 × 10^6^ CFU/mL reported by Wan et al. [37]. This study utilized the enterotoxigenic *Escherichia coli* (ETEC) F4ac (K88ac) strain, a well-recognized pathotype that causes porcine post-weaning diarrhea. This strain expresses F4ac (K88ac) fimbriae, a critical virulence factor that mediates specific adhesion to porcine intestinal epithelial cells [23,38]. The ETEC strain was cultured in antibiotic-free Luria-Bertani (LB) medium at 37 °C for 4 h, 6 h, or 8 h to obtain bacterial suspensions with different growth states. Bacterial concentration was converted from OD_600_ readings following based on the following formula: CFU/mL = k × OD_600_ × D, where k represents the fixed conversion coefficient, and D stands for the dilution factor of bacterial liquid. The multiplicity of infection (MOI, bacteria-to-cell ratio) was set to 10 for all infection groups [39]. Prior to bacterial treatment, the culture medium was replaced with antibiotic-free complete medium to eliminate antibiotic interference. A unified pretreatment protocol was applied for all experimental groups: IPEC-J2 cells were incubated with Ct-ELNs first, followed by ETEC challenge. During ETEC induction, culture conditions were strictly regulated to avoid excessive bacterial proliferation. After stimulation, cell monolayers were thoroughly rinsed with sterile PBS to eliminate non-adherent bacteria.

### 2.7. RNA Extraction and Quantitative Real-Time PCR (qPCR)

Cells were assigned to four experimental groups: a normal control group, a Ct-ELNs single-treatment group, a 1 × 10^6^ CFU/mL ETEC single-treatment group, and a 1 × 10^6^ CFU/mL ETEC + Ct-ELNs pretreatment group. After corresponding treatments, cells were incubated for an additional 6 h before sample collection. Total RNA was isolated using a commercial RNA extraction kit (TaKaRa Bio Inc., Kusatsu, Shiga, Japan) in accordance with the manufacturer’s instructions. RNA integrity and purity were assessed via agarose gel electrophoresis, and RNA concentration was quantified prior to reverse transcription. Complementary DNA (cDNA) was synthesized using MonScript™ RTIII Super Mix with dsDNase (one-step) (Monad Biotech Co., Ltd., Wuhan, Hubei, China). Quantitative real-time PCR (qPCR) was performed with Mon-Amp™ SYBR^®^ Green qPCR Mix on a Bio-Rad real-time PCR system (CT003142, Bio-Rad Laboratories, Hercules, CA, USA). No-template control and no-reverse-transcription control were included in each qPCR run to exclude non-specific amplification and genomic DNA contamination. Primer amplification efficiency and amplicon specificity were validated by melting curve analysis and agarose gel electrophoresis of PCR products, with detailed amplicon size information provided in the Table S1. Relative gene expression levels were calculated using the 2^−ΔΔCt^ method [40], with *β-actin* selected as the internal reference gene for normalization. Consistent with the experimental setup and the previous literature, *β-actin* exhibited stable transcriptional expression across all treatment groups in the present study [41,42]. All primer sequences used in this assay are listed in Table S1.

### 2.8. Cellular Protection

To investigate the effect of Ct-ELNs on cell viability after ETEC-induced damage to IPEC-J2 cells, dead/live staining was performed using a Calcein-AM/PI kit (Beyotime Biotechnology, Shanghai, China) following incubation, according to the product manual. Briefly, Calcein-AM and PI were added to the samples and incubated in the dark at 37 °C for 30 min. Fluorescent images were observed and captured using a fluorescence microscope (Olympus Corporation, Tokyo, Japan, IX73), and cell survival rates were analyzed using Image J software (Version 1.54f, National Institutes of Health, Bethesda, MD, USA) based on the proportion of live cells.

### 2.9. Statistics Analysis

The NTA data were processed using Origin 2024 (OriginLab Corporation, Northampton, MA, USA). Statistical analyses were conducted using GraphPad Prism 10.5 (GraphPad Software, San Diego, CA, USA). Intergroup differences were evaluated by one-way analysis of variance (ANOVA) followed by Tukey’s post hoc multiple comparison test. All results are presented as mean ± standard error of the mean (SEM) from three independent replicates. Different lowercase letters above bars denote significant differences (*p* < 0.05), and groups with identical letters show no statistical difference.

## 3. Results

### 3.1. Extraction, Purification, and Characterization of Ct-ELNs

**Figure 2:**
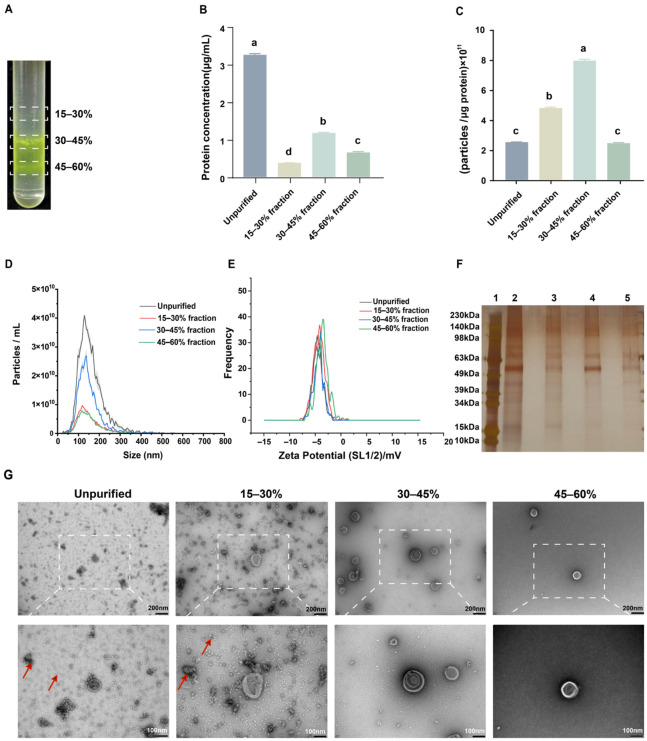
Characterization of safflower Ct-ELNs: (**A**) Schematic diagram of the distribution of Ct-ELNs in each gradient fraction after sucrose density gradient ultracentrifugation. (**B**) Total protein concentration of Ct-ELNs measured by BCA assay. The 30–45% fraction had the maximum protein content among purified components. (**C**) Sample purity analysis (particle number per microgram of protein; higher ratio indicates higher purity). The 30–45% density layer possessed the highest Ct-ELNs purity. (**D**) Nanoparticle tracking analysis (NTA) of safflower Ct-ELNs. The particle size of safflower Ct-ELNs is distributed between 80 and 180 nm. (**E**) Zeta potential distribution of Ct-ELNs fractions. (**F**) Protein content of safflower Ct-ELNs detected by silver staining. Safflower Ct-ELNs contained protein bands with multiple molecular weights, mainly ranging from 10 to 140 kDa. (**G**) Transmission electron microscopy (TEM) observation of safflower Ct-ELNs. Safflower Ct-ELNs exhibited a typical cup-shaped bilayer membrane structure; red arrows indicate non-Ct-ELNs. Compared with other fractions, the 30–45% safflower Ct-ELNs showed the cleanest background and the largest number of particles in the field of view (scale bar: 200 nm, 100 nm). Results were obtained from three independent experiments with three replicates per group (*n* = 3). Data are presented as mean ± standard error of the mean (mean ± SEM). Means with different letters (a–d) were significantly different at *p* < 0.05. Control: untreated cells (blank control); Unpurified: unpurified safflower Ct-ELNs; 15–30% fraction: 15–30% fraction safflower Ct-ELNs; 30–45% fraction: 30–45% fraction safflower Ct-ELNs; 45–60% fraction: 45–60% fraction safflower Ct-ELNs.

Ct-ELNs were purified by sucrose density gradient ultracentrifugation, and their distribution patterns in different gradient fractions were characterized (Figure 2A). Relying on differences in buoyant density, this approach enables efficient separation of Ct-ELNs from contaminants, which lays a foundation of high-purity samples for subsequent component screening and functional mechanism investigation.

BCA protein quantification results showed that the unpurified fraction had the highest total protein content; among all purified fractions, the 30–45% sucrose density fraction presented the maximum protein content (Figure 2B). Purity is a key indicator for evaluating the quality of extracellular vesicles (EVs). According to the EV purity criteria proposed by Webber et al., a particle-to-protein ratio exceeding 2 × 10^9^ particles per microgram of protein meets the standard for pure EVs [43]. After sucrose density gradient ultracentrifugation, the particle purity of all fractions was higher than this threshold. The 30–45% density layer exhibited the highest purity, confirming this layer as the main enrichment region of Ct-ELNs (Figure 2C).

NTA was performed to preliminarily determine the size distribution and concentration of particles in different sucrose layers. Through the NTA technique, the size distribution and the overall concentration of all the particles existing in different sucrose layers are shown in Figure 2D, respectively. Several peaks were observed in the graph, indicating highly concentrated particle populations with similar particle sizes and a distinct maximum (or mode). The curve corresponding to the 30–45% fraction was more uniform, suggesting that this fraction had a more consistent particle size distribution. All fractions presented negative surface charges, with main peaks ranging from −5 to −3 mV. The 15–30% fraction exhibited a zeta potential profile comparable to that of unpurified Ct-ELNs, whereas the 45–60% fraction showed slightly attenuated negative surface charges. The narrow peak distributions confirmed favorable colloidal homogeneity for all Ct-ELNs preparations (Figure 2E). SDS-PAGE was used to separate the proteins in each fraction via electrophoresis. Silver staining revealed the presence of proteins in Ct-ELNs, with the major proteins having a molecular weight range of 10 kDa to 140 kDa (Figure 2F).

It should be noted that particle size distribution and concentration determined by NTA alone cannot fully confirm the presence of Ct-ELNs in a sample. Thus, we further examined the morphology of particles and other present impurities in different layers by electron microscopy. Figure 2G demonstrated the presence of Ct-ELNs in all the fractions, which had typical cup-shaped bilayer membrane structure. In addition, TEM showed that that prior to purification, impurities were abundant in the Ct-ELNs (indicated by red arrows in Figure 2G). Following sucrose density ultracentrifugation purification, these impurities further enriched in the 15–30% fractions. This fraction exhibits high impurity content—a finding consistent with previous reports [44]. Compared with other fractions, the 30–45% safflower Ct-ELNs showed the cleanest background and the largest number of particles in the field of view.

We noted that minor fluctuations in particle and protein yield existed across plant batches, which may be attributed to slight differences in leaf growth status, harvest time, and environmental conditions; all functional biological experiments were performed using the same batch of Ct-ELNs to eliminate batch interference (Table S2).

To sum up, the present results demonstrated that sucrose density gradient ultracentrifugation enables the efficient acquisition of high-purity Ct-ELNs from the 30–45% sucrose fraction. This fraction is verified as the optimal component for Ct-ELNs purification in this study [45,46,47].

### 3.2. Cytotoxicity of Ct-ELNs on IPEC-J2 Cells

**Figure 3:**
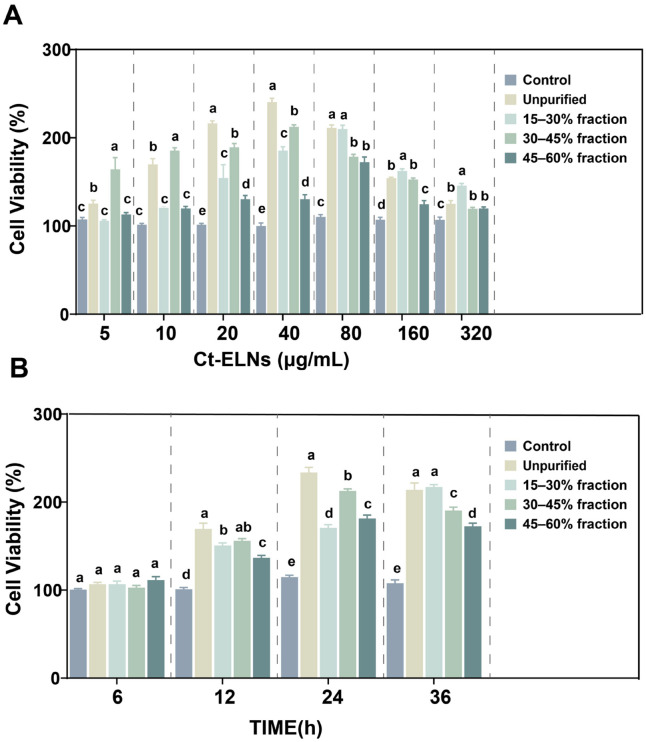
Effect of safflower Ct-ELNs on the viability of IPEC-J2 cells evaluated by the CCK-8 assay: (**A**) IPEC-J2 cells were co-incubated with safflower Ct-ELNs at concentrations of 5, 10, 20, 40, 80, 160, and 320 μg/mL for 24 h. (**B**) IPEC-J2 cells were treated with 40 μg/mL safflower Ct-ELNs for 6, 12, 24, and 36 h, respectively. Results were obtained from three independent experiments with three replicates per group (*n* = 3). Means with different letters (a–e) were significantly different at *p* < 0.05. Data are presented as mean ± standard error of the mean (mean ± SEM). Control: untreated cells (blank control); Unpurified: unpurified safflower Ct-ELNs; 15–30% fraction: 15–30% fraction safflower Ct-ELNs; 30–45% fraction: 30–45% fraction safflower Ct-ELNs; 45–60% fraction: 45–60% fraction safflower Ct-ELNs.

Before evaluating the regulatory effects of Ct-ELNs on IPEC-J2 cells, cell viability was detected via the CCK-8 assay under different treatment concentrations and incubation durations. As shown in Figure 3A, compared with the untreated control group, all four Ct-ELNs fractions with different purities exhibited no obvious cytotoxicity and improved cell viability in a protein-concentration-dependent manner (*p* < 0.05). At the protein concentration of 5 μg/mL, the 30–45% fraction produced a significant improvement in cell metabolic activity, indicating that this fraction possessed superior biological activity. When the concentration increased to 40 μg/mL, the metabolic-promoting effects of all fractions reached the peak, with significant differences observed among groups.

As illustrated in Figure 3B, Ct-ELNs treatment significantly enhanced the viability of IPEC-J2 cells in a time-dependent manner (*p* < 0.05). The beneficial metabolic effect was observed at 12 h after Ct-ELNs treatment. When cells were treated with 40 μg/mL Ct-ELNs for 24 h, all fractions achieved the maximum improvement in cellular metabolic activity. The unpurified group showed the most prominent effect, while the 30–45% fraction exhibited the optimal activity among all purified fractions, confirming its superior biological performance. Accordingly, the optimal treatment condition was determined as a protein concentration of 40 μg/mL and an incubation time of 24 h, and the 30–45% sucrose density gradient fraction displayed the most significant effect on improving IPEC-J2 cell metabolic activity and viability.

All cellular treatments throughout this study were normalized to total protein concentration instead of standardized particle number. Notably, different sucrose gradient fractions exhibit disparate particle-to-protein ratios and impurity profiles, such that equivalent protein dosages contain unequal amounts of vesicles and variable levels of co-extracted soluble contaminants. Accordingly, inter-fraction comparisons reported herein should be interpreted as preliminary results constrained by this normalization limitation.

### 3.3. In Vitro Cellular Uptake Profiles of Ct-ELNs in IPEC-J2 Cells

**Figure 4:**
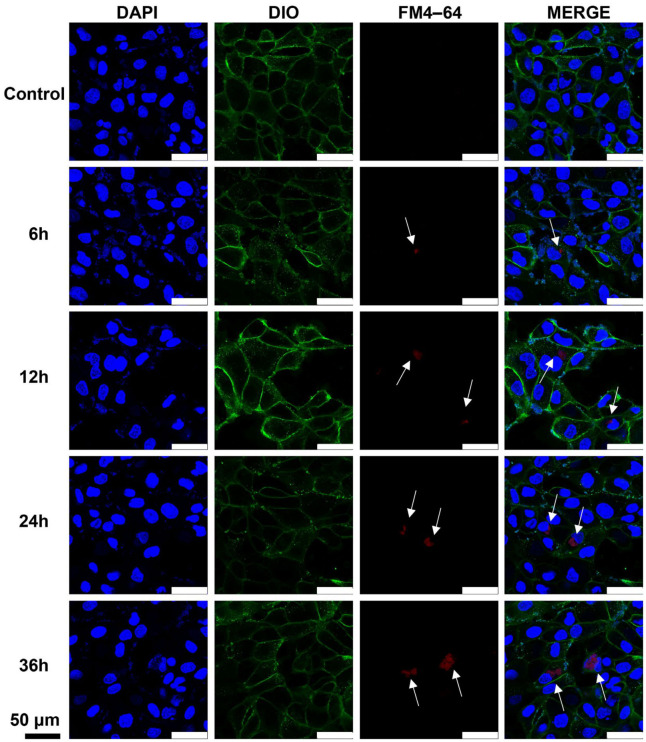
Confocal fluorescence microscopy images showing the uptake of safflower Ct-ELNs by IPEC-J2 cells: IPEC-J2 cells were incubated with FM4-64 (the double-membrane structure of 30–45% fraction of safflower Ct-ELNs emits strong red fluorescence upon binding with FM4-64)-labeled safflower Ct-ELNs for 6, 12, 24, and 36 h. IPEC-J2 cell nuclei were labeled with DAPI (blue); cell membranes were labeled with DIO (green); and 30–45% fraction of safflower Ct-ELNs were labeled with FM4-64 (red). White arrows indicate FM4-64-labeled 30–45% fraction of safflower Ct-ELNs. Scale bar = 50 μm. Control: untreated cells (blank control); 6~36 h: cells treated with 30–45% fraction safflower Ct-ELNs.

To visualize the dynamic process of Ct-ELNs endocytosis, IPEC-J2 cells were exposed to FM4-64 (FM4-64 possesses a lipophilic tail that can bind to Ct-ELNs with a bilayer membrane structure and exhibit intense red fluorescence)-labeled 30–45% fraction of Ct-ELNs with red fluorescence for 6, 12, 24, and 36 h and examined by confocal microscopy. No red fluorescent signal was detected in the control group, while red fluorescence gradually appeared in the treatment group with the extension of incubation time. A small amount of punctate red signals (white arrows) was observed at 6 h, indicating the initiation of Ct-ELNs uptake by cells. The red signals increased at 12 h, were significantly enhanced and diffused into the cytoplasm at 24 h. These results demonstrate that Ct-ELNs can be effectively taken up by IPEC-J2 cells in a time-dependent manner, with continuous accumulation within 24–36 h (Figure 4 and Figure S4). Combined with the negative control results in Figure S3, we ruled out the interference of free FM4-64 dye and confirmed that FM4-64 fluorescence suggests time-dependent association and likely cellular uptake of labeled Ct-ELNs by IPEC-J2 cells. In addition, intact cell morphology and clear cell nuclei without obvious apoptosis or shrinkage are observed throughout the experiment, further confirming that Ct-ELNs exert no cytotoxicity on IPEC-J2 cells and possess favorable biocompatibility.

### 3.4. Transcriptional Regulatory Effects of Ct-ELNs on ETEC-Stimulated IPEC-J2 Cells

**Figure 5:**
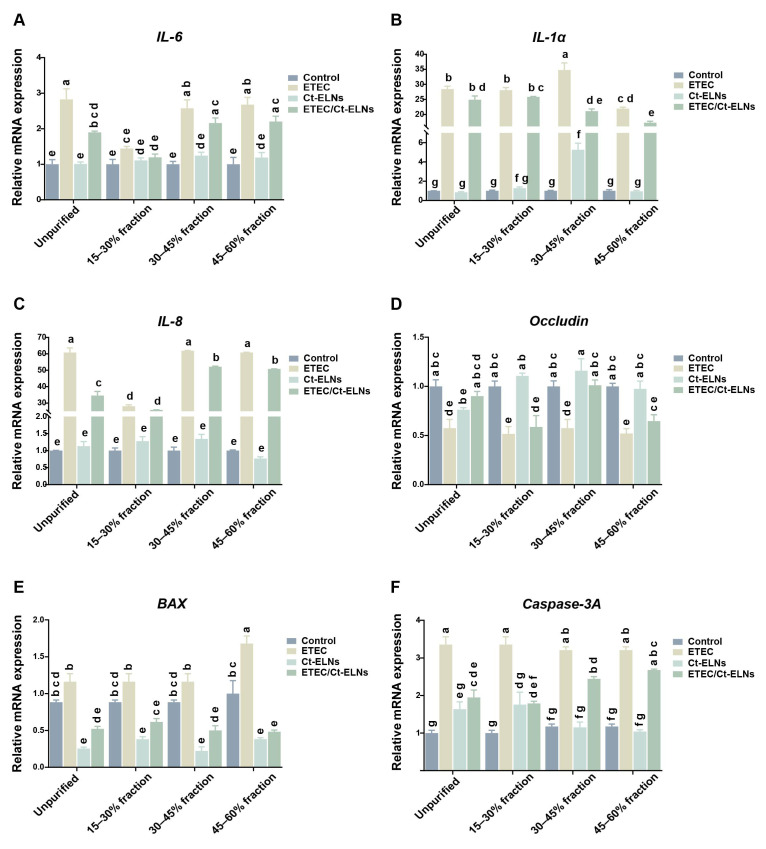
Effects of Ct-ELNs-enriched fractions on mRNA expression of inflammatory, barrier-related, and apoptosis-related genes in IPEC-J2 cells under ETEC challenge: (**A**) The relative mRNA expression of pro-inflammatory cytokine Interleukin-6 (*IL-6*); (**B**) he relative mRNA expression of pro-inflammatory cytokine Interleukin-1 alpha (*IL-1α*); (**C**) The relative mRNA expression of pro-inflammatory cytokine Interleukin-8 (*IL-8*); (**D**) The relative mRNA expression of tight junction protein (*Occludin*); (**E**) The relative mRNA expression of pro-apoptotic gene BCL-2-associated X protein (*BAX*); (**F**) The relative mRNA expression of apoptosis executor cysteine-dependent aspartate-specific protease-3A (*Caspase-3A*). Data are presented as the mean ± standard error (*n* = 3). Different lowercase letters (a–g) indicate significant differences among the values (*p* < 0.05). All values represent relative mRNA expression normalized to the reference gene *β-actin*. Unpurified: unpurified safflower Ct-ELNs; 15–30% fraction: 15–30% fraction safflower Ct-ELNs; 30–45% fraction: 30–45% fraction safflower Ct-ELNs; 45–60% fraction: 45–60% fraction safflower Ct-ELNs. Control: untreated cells (blank control); ETEC: cells treated with enterotoxigenic *Escherichia coli* (ETEC) alone; Ct-ELNs: cells treated with safflower Ct-ELNs alone; ETEC/Ct-ELNs: safflower Ct-ELNs treatment group combined with ETEC stimulation.

The optimal ETEC incubation time for establishing an inflammatory injury model in IPEC-J2 cells was determined according to Figure S1. ETEC infection impairs intestinal epithelial mucosal barrier function mainly by suppressing the expression of mucins and antimicrobial proteins and disrupting the distribution of tight junction proteins [48]. Therefore, this study explored the protective effects of Ct-ELNs by detecting their regulatory effects on ETEC-induced changes in inflammatory, apoptotic, and barrier-related gene expression in IPEC-J2 cells. As shown in Figure 5, ETEC infection significantly upregulated the mRNA levels of pro-inflammatory genes including *IL-6*, *IL-1α*, and *IL-8*, downregulated the mRNA expression of the tight junction gene *Occludin*, and activated cellular apoptosis, as evidenced by the increased transcription of *BAX* and *Caspase-3A*. No significant differences in the expression of the above genes were detected between the single Ct-ELNs treatment group and the blank control group. Compared with the ETEC model group, Ct-ELNs modulated ETEC-induced expression of inflammation-related genes in IPEC-J2 cells and partially restored ETEC-induced downregulation of *Occludin* mRNA, suggesting a possible effect on barrier-related gene expression (*p* < 0.05). The 30–45% sucrose fraction exhibited similar performance in modulating ETEC-induced expression of inflammation-related genes in IPEC-J2 cells as unpurified Ct-ELNs but with weaker efficacy, which failed to significantly reduce *IL-6* mRNA expression. Furthermore, all Ct-ELNs fractions remarkably inhibited the transcription of pro-apoptotic genes relative to the ETEC group (*p* < 0.05).

### 3.5. Fluorescence Staining Evaluation of Ct-ELNs on IPEC-J2 Cell Viability

**Figure 6:**
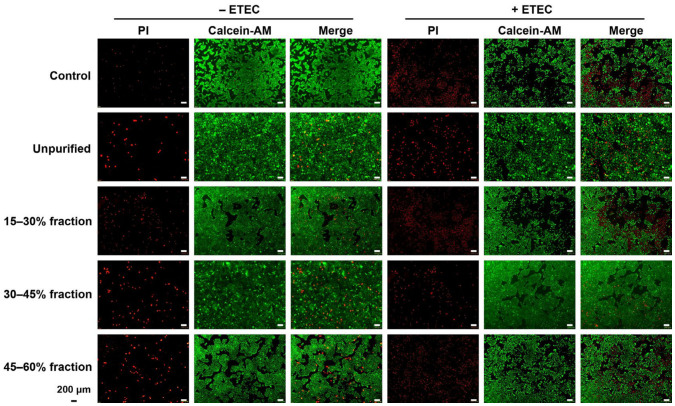
Fluorescence micrographs of protective effects in IPEC-J2 cells: Cell death detected via the Live/Dead assay. Dead cells were labeled with PI (red); living cells were labeled with Calcein-AM (green). Scale bar = 200 μm. (Scale bar = 200 μm. Control: untreated cells (blank control,); Unpurified: unpurified safflower Ct-ELNs; 15–30% fraction: 15–30% fraction safflower Ct-ELNs; 30–45% fraction: 30–45% fraction safflower Ct-ELNs; 45–60% fraction: 45–60% fraction safflower Ct-ELNs. −ETEC: cells without enterotoxigenic *Escherichia coli* (ETEC) treatment; +ETEC: safflower Ct-ELNs treatment group combined with ETEC stimulation.

To intuitively evaluate the in vitro regulatory effects of Ct-ELNs on IPEC-J2 cells and clarify their proliferation-promoting effect on normal cells as well as protective effect against ETEC-induced cell injury, Calcein-AM/PI double fluorescent staining was adopted in this study. Living cells were labeled with green fluorescence, while dead cells and late apoptotic cells were labeled with red fluorescence. The survival levels of cells in each group were observed and quantified under a fluorescence microscope. Under the normal physiological state of IPEC-J2 cells (–ETEC), the area of green fluorescence (live cells) in the Ct-ELNs pretreatment groups was significantly larger than that in the control group, indicating that Ct-ELNs exert pro-proliferative effects. Among these groups, the green fluorescence intensity of the unpurified safflower Ct-ELNs and 30–45% fraction safflower Ct-ELNs treatment groups (unpurified, 30–45% fraction) was higher than that of the 15–30% fraction safflower Ct-ELNs and 45–60% fraction safflower Ct-ELNs treatment groups (15–30% fraction; 45–60% fraction), further confirming that the 30–45% fraction exhibits superior effects in improving cell viability (Figure 6 and Figure S5).

In ETEC-injured IPEC-J2 cells (+ETEC), the model group displayed extensive black void areas, which represented cell death and cellular shedding. In contrast, Ct-ELNs-treated groups presented only a small number of red-stained dead cells and showed a markedly increased green fluorescence area relative to the ETEC model group. Combined with the RT-qPCR data of apoptosis-related genes and Live/Dead staining phenotypes, these results indicate that Ct-ELNs alleviate ETEC-induced cellular damage and suppress the expression of apoptosis-associated genes. Among different Ct-ELNs treatments, unpurified safflower Ct-ELNs and the 30–45% sucrose fraction yielded significantly larger green fluorescence areas and fewer red fluorescence signals compared with the 15–30% and 45–60% fractions. Furthermore, the 30–45% fraction group exhibited negligible large-scale black voids and the most intact cellular morphology (Figure 6 and Figure S5), intuitively demonstrating that this Ct-ELNs fraction possesses cytoprotective capacity. This phenotypic outcome is consistent with the RT-qPCR results showing decreased inflammatory factor expression and elevated levels of apoptosis-related and tight junction genes (Figure 5). Collectively, morphological and fluorescence evidence validates that Ct-ELNs reduced the expression of apoptosis-related genes and improved cell survival in the Live/Dead assay.

## 4. Discussion

In this study, we established a method for the extraction and purification of Ct-ELNs from safflower leaves via sucrose density gradient ultracentrifugation. Further functional analyses verified the in vitro cellular uptake of Ct-ELNs by IPEC-J2 cells and indicated that Ct-ELNs increased cellular metabolic activity. Moreover, when administered as a pretreatment agent, Ct-ELNs effectively alleviated cell damage induced by ETEC. Notably, the efficacy of Ct-ELNs-containing fractions was closely associated with the purity of Ct-ELNs. Among all purified fractions separated by density gradient ultracentrifugation, the 30–45% sucrose gradient component showed optimal regulatory performance: it powerfully modulated ETEC-triggered expression of inflammation-related genes in IPEC-J2 cells and exhibited the strongest ability to recover cell viability (Figure 3 and Figure 5). This result confirms that sucrose density gradient ultracentrifugation can effectively enrich functionally active vesicles.

Currently, commonly used extraction methods for PELNs include differential centrifugation, sucrose density gradient centrifugation, size exclusion chromatography (SEC), ultrafiltration, and immunoaffinity isolation microfluidics technology. A comparative analysis of different extraction methods is provided in Table S3 [49,50,51,52,53]. Among these, sucrose density gradient centrifugation possesses the advantages of high separation efficiency, low vesicle deformation rate, and high product purity [48]. Although sucrose density gradient ultracentrifugation has been applied for the purification of PELNs, the experimental evidence for determining the optimal fraction selection criteria remains insufficient [54,55,56]. Based on the above, the present study employed sucrose density gradient centrifugation to isolate Ct-ELNs from safflower leaves.

In this study, we established a separation method for safflower leaf Ct-ELNs based on sucrose density gradient ultracentrifugation. Characterization results from TEM and NTA showed that the obtained Ct-ELNs exhibited a typical disc-like morphology with uniform size distribution, and their structural characteristics were consistent with the typical features of PELNs [57,58], as well as with CDNVs (*Carthamus tinctorius* L. stigma-derived nanovesicles) isolated from safflower filaments [32]. Meanwhile, sucrose density gradient ultracentrifugation enabled better purification and enrichment of Ct-ELNs: TEM images (Figure 2G) revealed that Ct-ELNs obtained from the 30–45% fraction had a purer background and a greater number of particles per field of view. According to NTA results combined with the criteria for evaluating PELNs purity proposed by Webber et al. [43], the 30–45% fraction showed more uniform particle size distribution and the highest purity (Figure 2C,D). Results from BCA protein quantification and the protein SDS-PAGE electrophoresis (Figure 2B,F) also indicated that Ct-ELNs from the 30–45% fraction had higher protein content. Comprehensive characterization methods supported that the 30–45% fraction contained Ct-ELNs with the highest particle concentration, purity, and protein content. In subsequent experiments, we further demonstrated that Ct-ELNs from this 30–45% fraction possessed the most significant biological activity.

In mammalian systems, Cluster of Differentiation 9 (CD9) and Cluster of Differentiation 63 (CD63) are well-recognized markers for mammalian cell-derived exosomes (MEs) and also serve as the key criteria for exosome identification via Western blotting [59]. In contrast, due to the diversity of plant species, there is currently a lack of universal protein markers applicable to the PELNs. Although plant exosome-derived Tetraspanins (TET) identified in model plants—such as AtTET8/AtTET9 in Arabidopsis thaliana and OsTET7/OsTET13 in Oryza sativa—exhibit structural homology with CD63 [60,61], it remains to be verified whether such proteins can act as reliable markers for Ct-ELNs in non-model plants like safflower. Furthermore, for the Western blot analysis of Ct-ELNs, distinct specific markers or corresponding antibodies are still unavailable, which impedes the standardized identification of this system.

The cellular uptake of PELNs is a crucial process for their implementation of drug delivery functions and protective effects in an in vitro model. Results in Figure 4 showed that when red fluorescently labeled Ct-ELNs were co-cultured with IPEC-J2 cells, the intensity of red fluorescence in the cytoplasm gradually increased with the prolongation of co-culture time, suggesting that IPEC-J2 cells could internalize and uptake Ct-ELNs. Additionally, the fluorescence distribution pattern was consistent with the previously reported intracellular distribution characteristics of PELNs [62,63]. The endocytic mechanisms of mammalian exosomes include membrane fusion, lectin-dependent pathways, phagocytosis-mediated endocytosis, and other modes [64,65,66]. The specific internalization mechanism of Ct-ELNs in IPEC-J2 cells has not been clarified in this study. Future research can draw on the classical absorption pathways of MEs and conduct in-depth exploration of their uptake routes through specific inhibitor experiments.

The IPEC-J2 cell line is a well-recognized in vitro model for studying cytotoxicity and drug delivery effects [67]. The CCK-8 assay primarily reflects cellular metabolic activity and cell viability. Results of the CCK-8 assay (Figure 3) showed that low concentrations of Ct-ELNs significantly improved the metabolic activity and viability of IPEC-J2 cells in a concentration- and time-dependent manner. This result was consistent with the previously reported effects of CDNVs derived from safflower stigmas on HUVEC cells. The IPEC-J2 cell line is a well-recognized in vitro model for studying cytotoxicity and drug delivery effects [32]. All in vitro biological assays in the present study standardized Ct-ELNs treatment dosages according to total protein concentration. Ct-ELNs fractions obtained through sucrose density gradient centrifugation exhibit inherent heterogeneity in particle–protein ratios and impurity profiles, resulting in discrepant actual nanoparticle abundances even among groups with equivalent total protein levels. The total protein concentrations and particle count of individual fractions are summarized in Supplementary Table S2 to explicitly illustrate these inter-fraction differences. Notably, single-parameter quantification relying solely on total protein content cannot eliminate systematic errors derived from varying particle numbers across fractions, which compromises experimental data consistency and intergroup comparability. This technical bottleneck is prevalent in research on plant-derived nanovesicles. Future studies will adopt a dual-calibration strategy combining protein quantification and particle counting to optimize dosage normalization, thereby further enhancing experimental reproducibility and strengthening the robustness of relevant research conclusions.

Experimental results indicated that there were differences in the effects on cell viability among Ct-ELNs fractions with different densities isolated from safflower leaves. This variation was consistent with the characterization results of NTA and TEM. The 30–45% fraction showed the most prominent effect on improving cell viability. This further confirms that this fraction is the optimal purified component. It is worth noting that the pro-proliferative effects of plant-derived PELNs exhibit species specificity. PELNs derived from blueberries do not enhance the viability of Caco-2 cells [36,62], and citrus-derived PELNs also exert no significant influence on the survival rate of Caco-2 cells [36], whereas Ct-ELNs present a remarkable pro-proliferative effect on IPEC-J2 cells. The differences in the effects of PELNs from the aforementioned species on cells may be attributed to the differences in cell types (intestinal epithelial cells vs. colon cancer cells) or the variations in bioactive components contained in PELNs from different plants.

Pro-inflammatory cytokines (such as *IL-1α*, *IL-6*, and *IL-8*) are core effector molecules in the inflammatory response, participating in the initiation and progression of inflammation, thereby regulating the inflammatory response and disease development [68,69]. For example, they play a key role in IPEC-J2 cell inflammation induced by *Escherichia coli* [70,71,72]. In the inflammatory response of cells induced by ETEC, ETEC infection led to extremely significant increases in the mRNA transcription levels of *IL-1α*, *IL-6*, and *IL-8* in IPEC-J2 cells (Figure 5). However, pretreatment with Ct-ELNs significantly downregulated the expression levels of inflammatory factor genes in ETEC-induced IPEC-J2 cells, indicating that Ct-ELNs exert a damage-repairing effect on IPEC-J2 cells. This effect is consistent with the cellular anti-inflammatory effects of various PELNs. For instance, in LPS-stimulated Raw264.7 macrophages, PELNs extracted from black nightshade fruits significantly reduced the expression of *IL-6* [73]. Similarly, PELNs derived from cabbage and red cabbage decreased the secretion of *IL-6*, *IL-1β*, and *COX-2* in LPS-stimulated Raw264.7 cells [73].

Cell death is an important component of the inflammatory response, including various forms such as apoptosis, necrosis, and pyroptosis [74]. Studies have shown that ETEC infection can upregulate the expression of genes such as *Caspase-3A* in IPEC-J2 cells. The regulation of cell apoptosis involves the balance of BCL-2-associated X protein (*BAX*). In IPEC-J2 cells, ETEC infection can upregulate the expression of the *BAX* gene, disrupting the normal balance and promoting the occurrence of apoptosis [70,75,76]. These findings are in line with our results (Figure 5). Compared with the normal control group, the ETEC group presented significantly elevated mRNA levels of *BAX* and *Caspase-3A*. By contrast, Ct-ELNs pretreatment downregulated the expression of these two genes in ETEC-challenged IPEC-J2 cells. These results suggest that Ct-ELNs alleviate ETEC-triggered cell damage and modulate the transcription of apoptosis-related genes. Immunofluorescence images in Figure 6 further revealed fewer dead cells in the Ct-ELNs pretreatment group, which agreed well with the gene expression profiles. The Ct-ELNs pretreatment group also contained more viable cells, consistent with the cell viability data obtained from the CCK-8 assay in Figure 3. These results demonstrate that Ct-ELNs improve cell viability under normal physiological conditions and protect IPEC-J2 cells against ETEC-induced injury. Similar effects have been reported for plant exosome-like nanoparticles (PELNs) from other plant species. For instance, PELNs from Hedyotis diffusa Willd. upregulated BAX expression and induced apoptosis in hepatocellular carcinoma Huh-7 cells, while improving the viability of normal WRL68 hepatocytes [77]. Likewise, green-tea-derived PELNs increased BAX expression in HepG2 and Huh-7 cells and enhanced the viability of normal L02 hepatocytes [78].

Crudely extracted Ct-ELNs may contain non-PELNs components, resulting in low purity (Figure 2C,F). After purification by sucrose density gradient ultracentrifugation, Ct-ELNs from different fractions exhibited differential regulatory effects, among which the 30–45% fraction showed more significant regulatory effects. However, further research is needed to comprehensively identify the relevant protein profiles involved. The results of this study indicate that Ct-ELNs can regulate gene expression in IPEC-J2 cells. However, the core bioactive substances within Ct-ELNs (including characteristic miRNAs, proteins, and lipid molecules) responsible for modulating ETEC-induced expression of inflammation-related genes in IPEC-J2 cells remain unidentified. Multi-omics approaches are required for further identification in subsequent studies.

Biological waste recycling represents a critical issue concerning environmental sustainability and economic benefits. As reported in the Food Waste Index Report 2024 released by the United Nations Environment Programme (UNEP), global food waste reached approximately 1.05 billion tons in 2022. Approximately 19% of food available to consumers is wasted at retail, foodservice, and household levels (https://wedocs.unep.org/20.500.11822/45230, accessed on 12 March 2026). Therefore, strategies for extracting high-value products from these waste streams are of great practical significance. Safflower leaves, as an abundant, low-cost, and underutilized biological resource, have great potential for conversion into high-value products. In this study, Ct-ELNs capable of modulating inflammation-related gene expression were successfully extracted from safflower leaves and purified via sucrose density gradient ultracentrifugation. The results revealed significant discrepancies in such regulatory effects across fractions separated at different sucrose concentrations, which may be attributed to variations in Ct-ELNs purity and abundance within each fraction. The above findings confirm that sucrose density gradient ultracentrifugation can effectively achieve the extraction and purification of Ct-ELNs.

The present study has several inherent limitations. First, all functional validations were performed using in vitro IPEC-J2 cell models, and in vivo animal experiments were not included to further confirm the protective efficacy of Ct-ELNs. Second, the conclusions regarding intestinal barrier integrity were only supported by the transcriptional expression of tight-junction-related genes, without complementary functional evaluations (e.g., TEER and FITC-dextran permeability assays) and protein-level validation of tight junction proteins. Third, apoptosis assessment in this study was limited to apoptosis-related gene quantification and Live/Dead staining. Classic apoptotic detection assays, including Annexin V/PI flow cytometry, TUNEL staining, and cleaved caspase protein quantification, were not performed. Future work will supplement these missing validations to obtain more comprehensive and robust evidence for the protective mechanisms of Ct-ELNs.

## 5. Conclusions

This study established a method to isolate and purify Ct-ELNs from safflower leaves using sucrose density gradient centrifugation. The 30–45% sucrose fraction yielded Ct-ELNs with high purity, typical bilayer structure, and uniform particle size. In vitro assays demonstrated that FM4-64 fluorescence signals indicated time-dependent cellular adhesion and internalization of labeled Ct-ELNs in IPEC-J2 cells. Moreover, Ct-ELNs enhanced cell viability and modulated ETEC-triggered expression of inflammation-related genes. This work offers a new way for value-added utilization of safflower leaves and enriches research on plant-derived ELNs.

All findings in this work are preliminary results obtained solely from in vitro cell experiments. Owing to the limitation of protein-normalized dosing, the differential bioactivities of different sucrose fractions cannot be fully attributed to intrinsic Ct-ELNs properties, as disparities in particle number and co-isolated soluble contaminants may contribute to the observed effects. Further investigations including mechanistic exploration, systematic safety evaluation, gastrointestinal digestion stability testing, and in vivo animal verification are urgently needed. Moreover, this study only adopted sucrose density gradient centrifugation for purification without comparative analysis against other conventional plant extracellular vesicle isolation approaches and failed to identify the core bioactive components inside Ct-ELNs via proteomics or multi-omics profiling. Follow-up studies will address the above deficiencies to advance the translational potential of safflower leaf-derived Ct-ELNs.

## Data Availability

The original contributions presented in this study are included in this Article/Supplementary Material. Further inquiries can be directed to the corresponding authors.

## Supplementary Materials

The following supporting information can be downloaded at: https://www.mdpi.com/article/10.3390/foods15142417/s1, Figure S1. Quality verification of total RNA and specificity identification of qPCR products; Figure S2. Establishment of an ETEC-induced epithelial injury model in IPEC-J2 cells; Figure S3. Confocal fluorescence microscopy images showing the uptake of safflower Ct-ELNs by IPEC-J2 cells; Figure S4. Time-lapse confocal microscopy tracking the internalization of 30–45% sucrose fractionated safflower Ct-ELNs in IPEC-J2 cells; Figure S5. Quantitative analysis of live and dead cell proportions based on Live/Dead fluorescence images. Table S1. Sequence and abbreviations of real-time PCR primers; Table S2. Median particle size, particle concentration, protein concentration, particle yield, and protein yield of Ct-ELNs isolated from different sucrose gradient fractions; Table S3. Principles, advantages, and disadvantages of different extraction techniques for PELNs. References [79,80,81,82,83,84,85,86,87,88,89] are cited in the supplementary materials.

## Abbreviations

The following abbreviations are used in this manuscript:

Ct-ELNs	*Carthamus tinctorius* L.-derived Exosome-like Nanoparticles
PELNs	Plant-derived Exosome-like Nanoparticles
EVs	Extracellular Vesicles
ETEC	Enterotoxigenic *Escherichia coli*
NTA	Nanoparticle Tracking Analysis
TEM	Transmission Electron Microscopy
*IL-6*	Interleukin-6
*IL-1α*	Interleukin-1alpha
*IL-8*	Interleukin-8
*BAX*	BCL-2-associated X protein
*Caspase-3**A*	Cysteine-dependent aspartate-specific protease-3A
*β-actin*	Beta-actin
