# Supplementary material for: Isolation, Characterization, and Anti-Inflammatory Effects of Carthamus tinctorius L. Leaf-Derived Exosome-like Nanoparticles in ETEC-Challenged IPEC-J2 Cells
Source: Foods. 2026 Jul 8;15(14):2417. doi: 10.3390/foods15142417 (PMC13407690; doi:10.3390/foods15142417)
Supplement: Supplementary file 1 [file foods-15-02417-s001.zip › foods-4356221-supplementary.pdf]

Figure S1

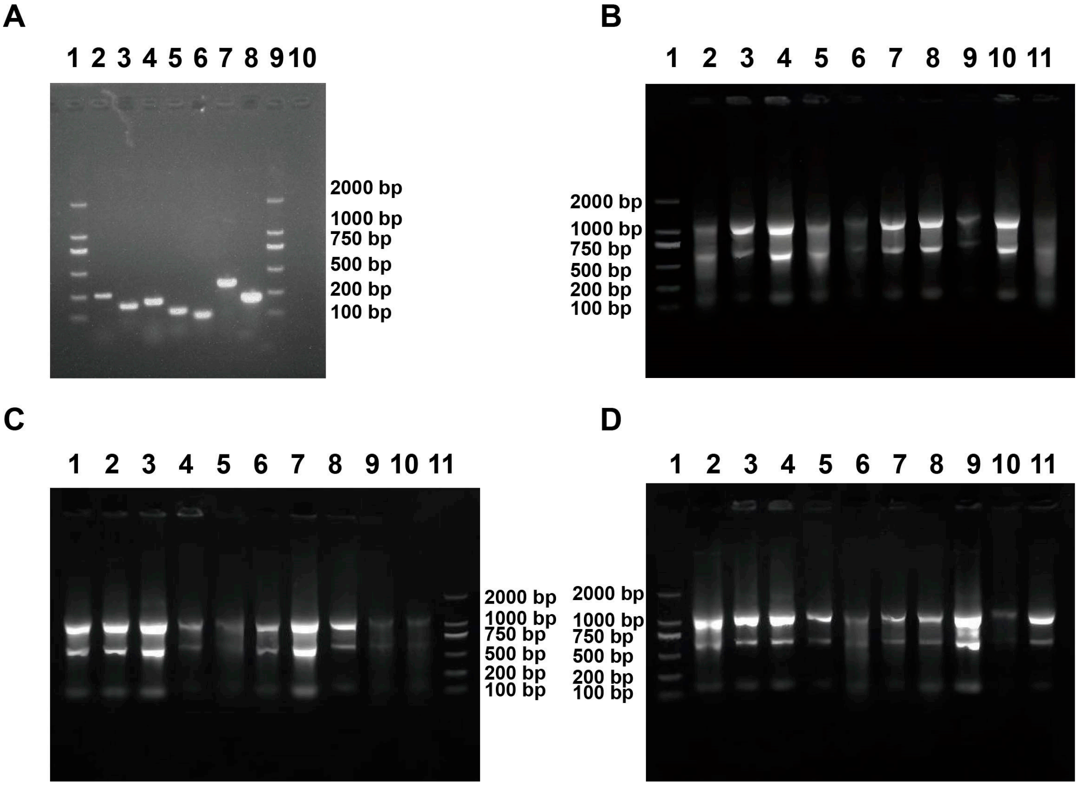

Figure S1. Quality verification of total RNA and specificity identification of qPCR products: **(A)** Agarose gel electrophoresis of qPCR amplified products. (1: DL2000 Marker, 2: *Occludin*, 3: *BAX*, 4: *IL-8*, 5: *IL-1α*, 6: *IL-6*, 7: *Caspase-3A*, 8: *β-actin*, 9: DL2000, 10: negative control, 7: empty vector). **(B-D)** Total RNA agarose gel electrophoresis. (**B and D** 1: DL2000 Marker, 2: Control: untreated cells (blank control,), 3: ETEC: only *Escherichia coli* (ETEC) treatment, 4: Unpurfied–ETEC: unpurified safflower Ct-ELNs, 5: 15–30% fraction-ETEC: 15–30% fraction safflower Ct-ELNs; 6: 30–45% fraction-ETEC: 30–45% fraction safflower Ct-ELNs; 7: 45–60% fraction-ETEC: 45–60% fraction safflower Ct-ELNs. 8: Unpurfied+ETEC: unpurified safflower Ct-ELNs, 9: 15–30% fraction-ETEC: 15–30% fraction safflower Ct-ELNs; 10: 30–45% fraction-ETEC: 30–45% fraction safflower Ct-ELNs; 11: 45–60% fraction-ETEC: 45–60% fraction safflower Ct-ELNs.) (**C**1: Control: untreated cells (blank control,), 2: ETEC: only Escherichia coli (ETEC) treatment, 3: Unpurfied–ETEC: unpurified safflower Ct-ELNs, 4: 15–30% fraction-ETEC: 15–30% fraction safflower Ct-ELNs; 5: 30–45% fraction-ETEC: 30–45% fraction safflower Ct-ELNs; 6: 45–60% fraction-ETEC: 45–60% fraction safflower Ct-ELNs. 7: Unpurfied+ETEC: unpurified safflower Ct-ELNs, 8: 15–30% fraction-ETEC: 15–30% fraction safflower Ct-ELNs; 9: 30–45% fraction-ETEC: 30–45% fraction safflower Ct-ELNs; 10: 45–60% fraction-ETEC: 45–60% fraction safflower Ct-ELNs. 11: DL2000 Marker.) –ETEC: cells without enterotoxigenic ETEC treatment; +ETEC: safflower Ct-ELNs treatment group combined with ETEC stimulation.

Figure S2

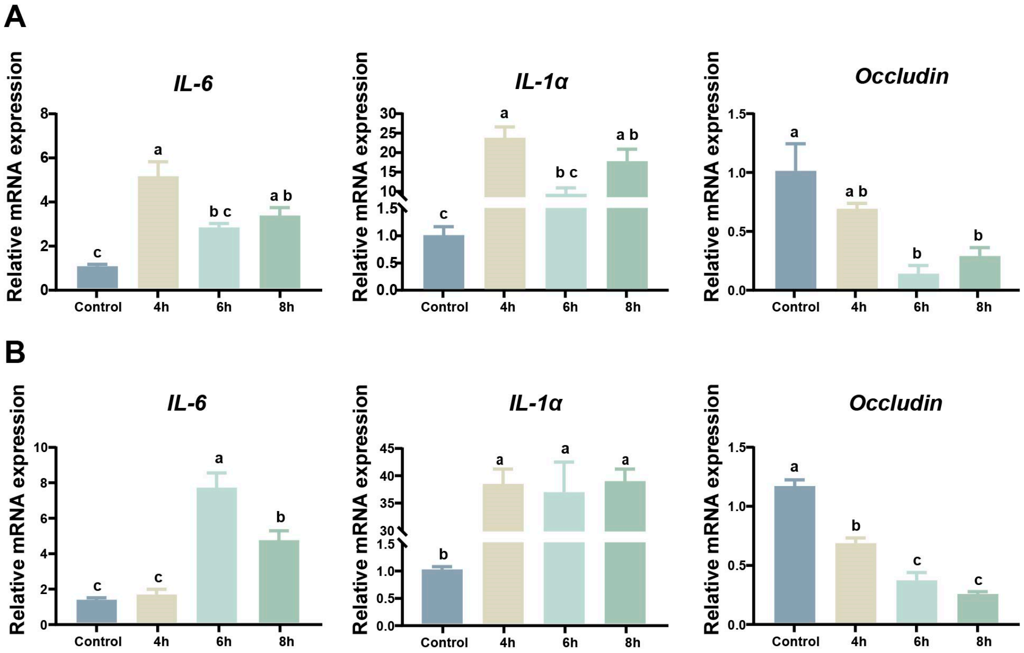

Figure S2. Establishment of an ETEC-induced epithelial injury model in IPEC-J2 cells: (**A**) Effects of different growth stages of ETEC on gene expression levels in IPEC-J2 cells. (**B**) Impact of ETEC treatment on IPEC-J2 cells at different time points on gene expression levels. Results were obtained from three independent experiments with three replicates per group ($n = 3$). Means with different letters (a-c) were significantly different at $p < 0.05$. Data are presented as mean ± standard error of the mean (mean ± SEM). All values represent relative mRNA expression normalized to the reference gene *β-actin*.

Figure S3

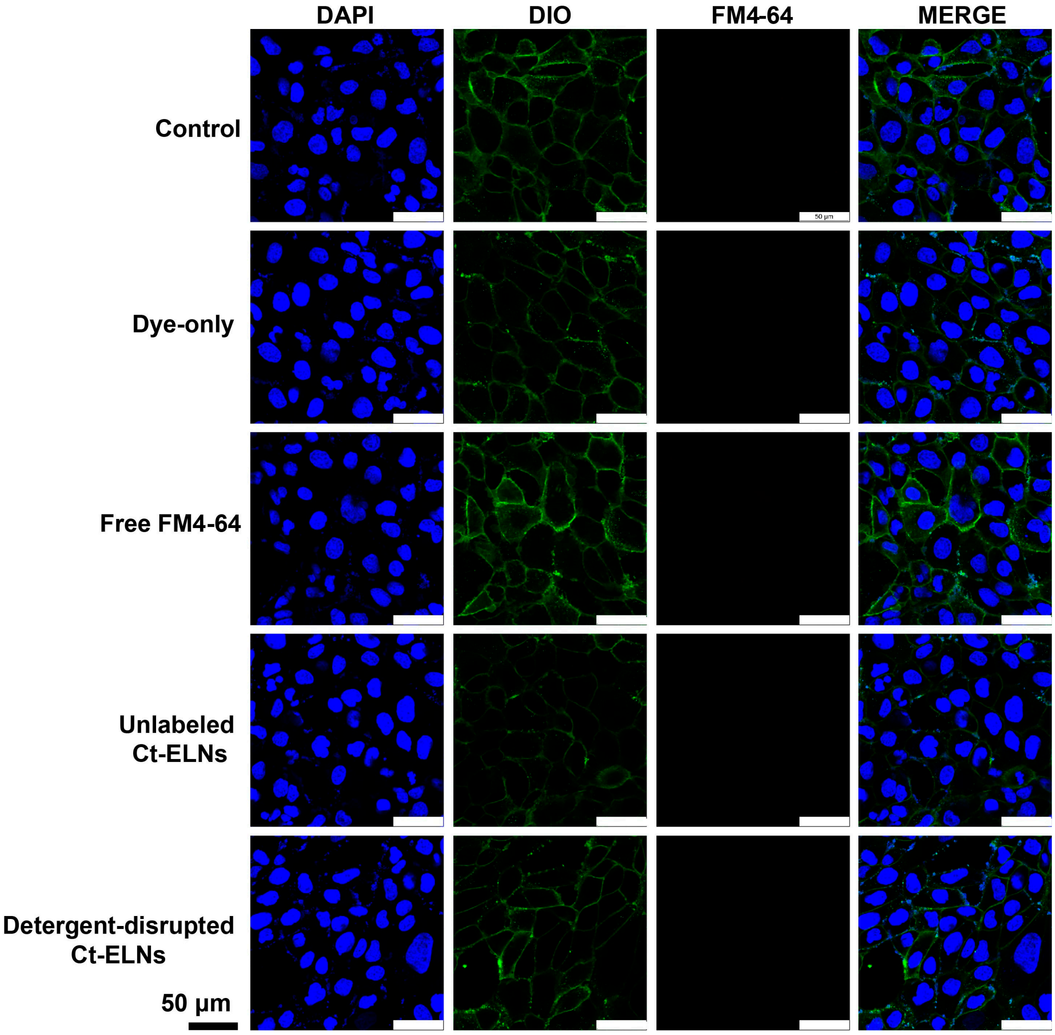

Figure S3. Confocal fluorescence microscopy images showing the uptake of safflower Ct-ELNs by IPEC-J2 cells. IPEC-J2 cell nuclei were stained with DAPI (blue), and cell membranes were labeled with DiO (green). Scale bar = 50 µm. Control: untreated cells (blank control); Dye-only control: cells incubated with free FM4–64for 24 h; Free FM4–64control: cells treated for 24 h with supernatant containing free FM4–64collected after ultracentrifugation; Unlabeled Ct-ELNs control: cells incubated with unlabeled Ct-ELNs for 24 h; Detergent-disrupted Ct-ELNs control: fluorescently labeled Ct-ELNs were pre-treated with 0.2% (v/v) Triton X-100 at room temperature for 15 min to fully disrupt lipid bilayer vesicles and release encapsulated FM4–64before cell incubation.

Figure S4

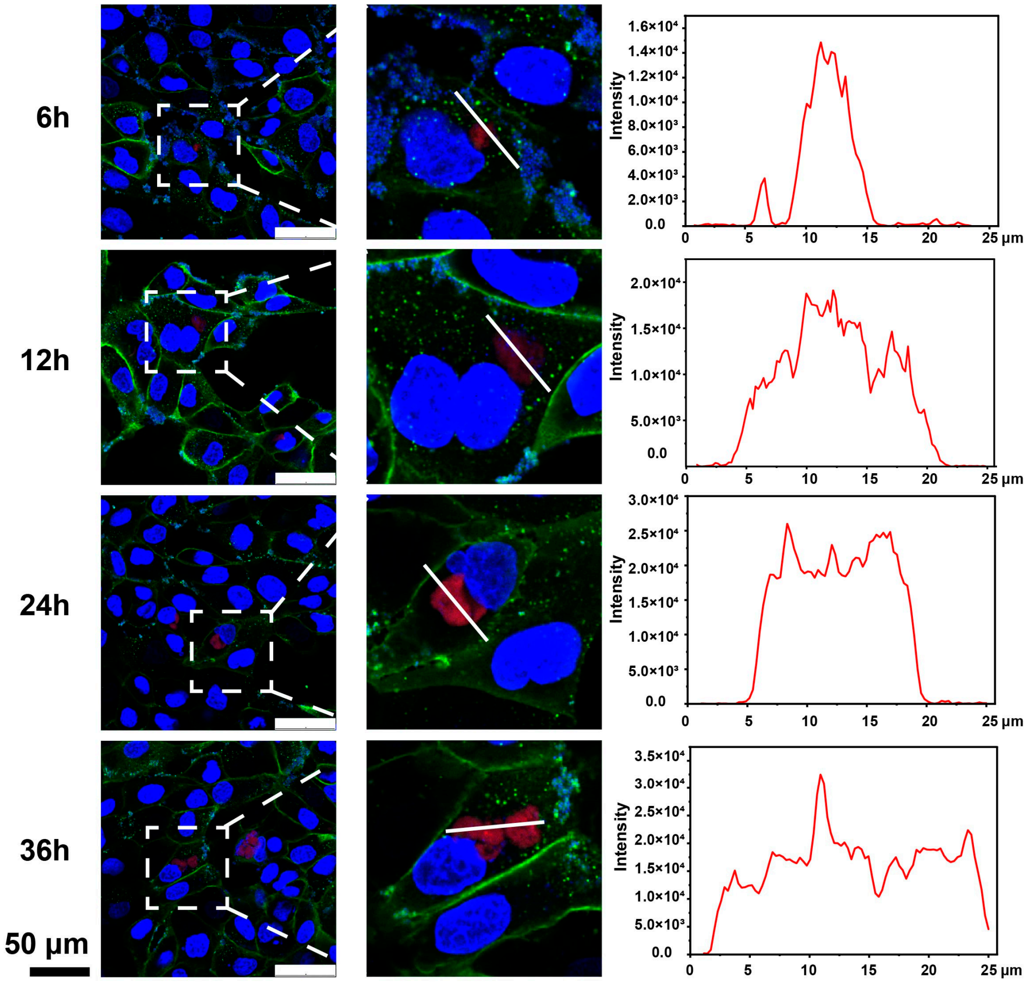

Figure S4. Time-lapse confocal microscopy tracking the internalization of 30–45% sucrose fractionated safflower Ct-ELNs in IPEC-J2 cells. Left panels: merged fluorescence images captured at 6, 12, 24 and 36 h post co-incubation (scale bar, 50 μm). Middle panels: magnified regions corresponding to the boxed areas in the left images. Right panels: quantitative fluorescence intensity profiles along the white lines drawn in the magnified fields.

Figure S5

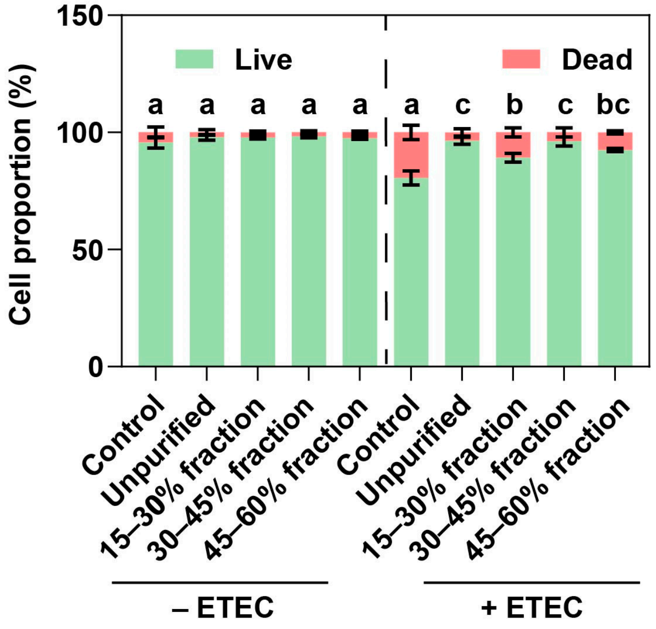

Figure S5. Quantitative analysis of live and dead cell proportions based on Live/Dead fluorescence images. Green bars represent viable Calcein-AM-positive cells, and red bars indicate dead cells stained with propidium iodide (PI). –ETEC represents cells cultured without ETEC stimulation, while + ETEC indicates epithelial cells challenged with ETEC to induce cell injury. Experimental groups include blank control, unpurified crude Ct-ELNs, and Ct-ELNs collected from 15–30%, 30–45%, and 45–60% sucrose density gradient fractions, respectively. The proportions of live and dead cells were quantified from confocal images using ImageJ software (version 1.54f, National Institutes of Health, Bethesda, MD, USA). Results were obtained from three independent experiments with three replicates per group ($n = 3$). Means with different letters (a-c) were significantly different at $p < 0.05$. Data are presented as mean ± standard error of the mean (mean ± SEM).

Table S1

Table S1. Sequence and abbreviations of real-time PCR primers

Gene name	Abbreviation	Product size (bp)	Primer sequence
Interleukin-6	*IL-6*	121	**F:** 5′-GGACGCCTGGAAGAAGAT-3′ **R:** 5′-TGAACCCAGATTGGAAG-3′
Interleukin-8	*IL-8*	189	**F:** 5′-AGAGTGGACCCCACTGTGAA-3′ **R:**5′-AAATTCTTGGGAGCCACGGA-3′
Interleukin-1 alpha	*IL-1α*	154	**F:** 5′-TAAGAATCTCAGAAACCCGAC-3′ **R:**5′-GGCTGATTTGAAGTAGTCCAT-3′
Occludin	*Occludin*	217	**F:** 5′-CAGGTGCACCCCTCCAGATTG-3′ **R:**5′-TGGACTTTCAAGAGGCCTGG-3′
BCL-2-associated X protein	*BAX*	178	**F:** 5′-CTACCAAGAAGTTGAGCGAGTGTC-3′ **R:** 5′-ACGGCTGCGATCATCCTCTG-3′
Cysteine-dependent aspartate-specific protease-3A	*Caspase-3A*	297	**F:** 5′-GGAATGCCATGTCGATCTGGT-3′ **R:** 5′-ACTGTCCGTCTCAATCCCAC-3′
Beta-actin	*β-actin*	196	**F:** 5′-CCCTGGCAAATGTACACACCT-3′ **R:** 5′-GAGCCGCGTGTGTGTAACTA-3′

Table S2

Table S2. Median particle size, particle concentration, protein concentration, particle yield, and protein yield of Ct-ELNs isolated from different sucrose gradient fractions

	Median particle size (nm)	Protein concentration (mg mL^{-1})	Concentration (particles mL^{-1})	Particle yield (particles g^{-1} fresh leaf)	Protein yield (mg/g fresh leaf)
Unpurified	144.4 ± 0.48	3.27±0.05	$1.4\times10^{11}\pm1.13\times10^{10}$	4.83×10^{9}	0.113
15–30% fraction	134.8 ± 0.98	0.38±0.01	$1.83\times10^{11}\pm3.3\times10^{9}$	4.42×10^{9}	0.0092
30–45% fraction	136.6 ± 0.22	1.18±0.03	$4.77\times10^{11}\pm8.82\times10^{9}$	1.15×10^{10}	0.0285
45–60% fraction	140.7 ± 0.12	0.66±0.03	$1.6\times10^{11}\pm3.33\times10^{9}$	3.86×10^{9}	0.0159

Note: All Ct-ELNs were isolated from 29 g of fresh safflower leaves for each independent experimental batch. Following sucrose density gradient fractionation, the unpurified crude pellet was resuspended in 1 mL sterile PBS,

whereas pellets from each sucrose gradient fraction were resuspended in 0.7 mL PBS. Particle yield (particles per gram fresh leaf) and total protein yield (mg per gram fresh leaf) were quantified to evaluate the high-value utilization potential of safflower leaf biomass. Slight inter-batch fluctuations in particle and protein yields were observed across separate leaf harvest batches in our preliminary trials, which could be ascribed to disparities in leaf maturity and growth conditions. To exclude batch-dependent confounding factors, all in vitro functional experiments were conducted using Ct-ELNs derived from one uniform leaf batch. Particle and protein yields were normalized based on fixed resuspension volumes and initial leaf weights. Accordingly, standard deviations were not separately presented for these normalized yield metrics, and the inherent variability of raw particle and protein concentration measurements is provided in the corresponding table columns.

Table S3

Table S3. Principles, advantages, and disadvantages of different extraction techniques for PELNs

Techniques	Principles	Advantages	Disadvantages	Examples
Differential centrifugation	Sedimentation coefficients [50]	Simple operation; free of contamination	Not for trace/valuable samples	Orange Juice [79]; Grapefruit [80]
Sucrose density gradient centrifugation	Density and particle size [49]	High product purity	Complicated procedures and time-consuming operations	Gardenia [81]; Ginger [82]
Size exclusion chromatography (SEC)	Particle size [83]	High product purity; intact ultrastructure	Purification yield limited by instrument constraints	Cabbage [84]
Ultrafiltration	Particle size and molecular weight [52]	Simple operation, low cost, high efficiency (capture)	Ultrafiltration membranes tend to clog with macromolecular substances	Arabidopsis Leaf [85]
Immunoaffinity isolation	Antigen-antibody specific binding [51]	High purity, excellent morphology, and high recovery rate	Saline concentration impacts biological activity	Arabidopsis [86]
Polymer Coprecipitation	Solubility reduction [87]	Lower equipment requirements and cost; high output	Low purity	Ginger [63]、*Lycium ruthenicum* Murray EVs [88]
Microfluidics technology	Size and Density [53]	Time-saving, experimental workflow	High demands on equipment	Rosa Damascena [89]
